# Identifying Potential Regions of Copy Number Variation for Bipolar Disorder

**DOI:** 10.3390/microarrays3010052

**Published:** 2014-02-28

**Authors:** Yi-Hsuan Chen, Ru-Band Lu, Hung Hung, Po-Hsiu Kuo

**Affiliations:** 1 Department of Public Health & Institute of Epidemiology and Preventive Medicine, College of Public Health, National Taiwan University, Taipei 100, Taiwan; E-Mails: r00849026@ntu.edu.tw (Y.-H.C.); hhung@ntu.edu.tw (H.H.); 2 Department of Psychiatry, College of Medicine & Hospital, National Cheng Kung University, Tainan 704, Taiwan; E-Mail: rblu@mail.ncku.edu.tw; 3 Research Center for Genes, Environment and Human Health, National Taiwan University, Taipei 100, Taiwan

**Keywords:** bipolar disorder, copy number variation, DNA pooling, filtering

## Abstract

Bipolar disorder is a complex psychiatric disorder with high heritability, but its genetic determinants are still largely unknown. Copy number variation (CNV) is one of the sources to explain part of the heritability. However, it is a challenge to estimate discrete values of the copy numbers using continuous signals calling from a set of markers, and to simultaneously perform association testing between CNVs and phenotypic outcomes. The goal of the present study is to perform a series of data filtering and analysis procedures using a DNA pooling strategy to identify potential CNV regions that are related to bipolar disorder. A total of 200 normal controls and 200 clinically diagnosed bipolar patients were recruited in this study, and were randomly divided into eight control and eight case pools. Genome-wide genotyping was employed using Illumina Human Omni1-Quad array with approximately one million markers for CNV calling. We aimed at setting a series of criteria to filter out the signal noise of marker data and to reduce the chance of false-positive findings for CNV regions. We first defined CNV regions for each pool. Potential CNV regions were reported based on the different patterns of CNV status between cases and controls. Genes that were mapped into the potential CNV regions were examined with association testing, Gene Ontology enrichment analysis, and checked with existing literature for their associations with bipolar disorder. We reported several CNV regions that are related to bipolar disorder. Two CNV regions on chromosome 11 and 22 showed significant signal differences between cases and controls (*p* < 0.05). Another five CNV regions on chromosome 6, 9, and 19 were overlapped with results in previous CNV studies. Experimental validation of two CNV regions lent some support to our reported findings. Further experimental and replication studies could be designed for these selected regions.

## 1. Introduction

Bipolar disorder (BPD) is a common mental disorder, which is characterized by the recurrence of manic and depressive episodes. The prevalence of BPD is around 1%–2%, and it accounts for a significant proportion of disease burden worldwide [1]. The estimated heritability of BPD is approximately 60%–85% [2]; however, the genetic determinants and its underlying pathogenesis are still not clear. In recent years, structural variations on DNA segments, in particular copy number variations (CNVs), have gained increasing attention in relation to complex traits. Array-based technologies enable high speed scanning of large numbers of CNVs. The identification of disease associated CNVs may help to explain some missing heritability that could not be explained by common SNPs (single nucleotide polymorphisms) [3]. Previously, a number of CNVs have been reported to be associated with different psychiatric disorders, such as schizophrenia, autism, and BPD [4,5,6,7]. 

One of the major challenges in conducting CNV studies at the genome-wide level comes from applying statistical approaches to detect associations. Although several statistical strategies are developed for the estimation of copy numbers from experimental data, there is no consensus for CNV calling [8]. The difficulties reside in estimating discrete values of the copy numbers using continuous signals calling from a set of markers, and simultaneously performing association testing between CNVs and phenotypic outcomes. In addition, different individuals might have varied breakpoints of defined CNV regions. Previously, a hidden Markov model (HMM) has often been applied to analyze CNV data [9,10]. HMM-based algorithms could simultaneously identify copy number status and the breakpoint of CNV regions for each individual. However, HMM-based methods are reported to have relatively high error rates in short CNV regions [8,11]. In addition, most of the diseases associated CNVs have only been found in a small number of subjects in previous CNV studies, and the reported CNV regions are usually with moderate effect size [12,13]. Due to relatively rare events and high genotyping costs, scanning CNVs at a genome-wide level in large-scale samples individually may not be cost-effective in the discovery phase. It is also difficult to perform association testing between CNV regions and diseases, and design follow-up experiments when the events are rare.

Recently, DNA pooling strategy was adopted to save genotyping cost [14,15]. A pool consists of a set of individuals, which may introduce noise and high variation into signal estimation. Nevertheless, with appropriate quality control and validation using individual genotyping in the later stages, the pooling strategy has been utilized in human genetics research [16], and could provide a more cost effective way to identify novel loci or chromosomal regions for complex traits [14,17]. So far, it is still a challenge to use pooling data for CNV detection. Recently, a HMM-based method was developed to analyze copy number status in DNA pooling data using Affymetrix SNP arrays [18]. Following experimental validation, the authors suggest that applying DNA pooling could help to discover more common CNV regions. However, this algorithm deals only with Affymetrix array-data. For genome-wide CNV array-data from other platforms, there is a need to develop more general filtering procedures to reduce noise and perform data analysis whilst using a DNA pooling strategy. We believe that by minimizing potential errors in CNV calling, the chance for correctly evaluating the relationships between CNVs and the trait of interest would be substantially increased. To our best knowledge, there are no genome-wide CNV studies that are conducted for BPD in Asian populations yet. The goal of the present study is to develop a series of filtering and data analysis procedures to identify potential CNV regions for BPD in a Han Chinese population using a DNA pooling strategy. 

## 2. Methods

### 2.1. Subjects, DNA Pooling Construction and Genotyping

We conducted a family study of mood disorders in Taiwan from 2008–2012. Recruitment and clinical characteristics of the participants are described in more detail elsewhere [19,20]. In brief, patients aged between 18 and 70 years and diagnosed with major depression disorder (MDD), bipolar I disorder (BPD-I), or bipolar II disorder (BPD-II) according to the DSM-IV (Diagnostic and Statistical Manual of Mental Disorders, fourth edition), were consecutively referred by psychiatrists in Taiwan. Independent healthy controls were recruited by methods of sending leaflets or “word of mouth” in the community. All of the controls were screened for mood disturbances and other major psychotic disorders. For every participant, we asked questions about ethnicity; only participants whose parents and grandparents are all Han Chinese were enrolled. The study and data collection procedures were approved by the Institutional Review Broad of all participating institutes and hospitals. All participants provided written informed consent after details of the study were fully illustrated.

Blood samples were taken to extract DNA for each individual. A total of 200 independent BPD-I patients and 200 healthy controls were selected with good quality DNA, and we randomly divided them into eight case and eight control groups (each with 25 subjects). DNA concentration and quality were twice checked using Quant-iT™ PicoGreen^®^ dsDNA Reagent and Kits (Invitrogen, Carlsbad, CA, USA). Equivalent amounts of DNA from each subject were mixed together to create eight case pools and eight control pools. For details of the experimental procedures and DNA pooling strategies please see elsewhere [21]. Whole-genome pooling genotyping was performed using Illumina HumanOmni1-Quad array with approximately one million markers including SNP and CNV probes. 

### 2.2. Quality Control and Filtering Procedures for CNV Analysis

**Figure 1:**
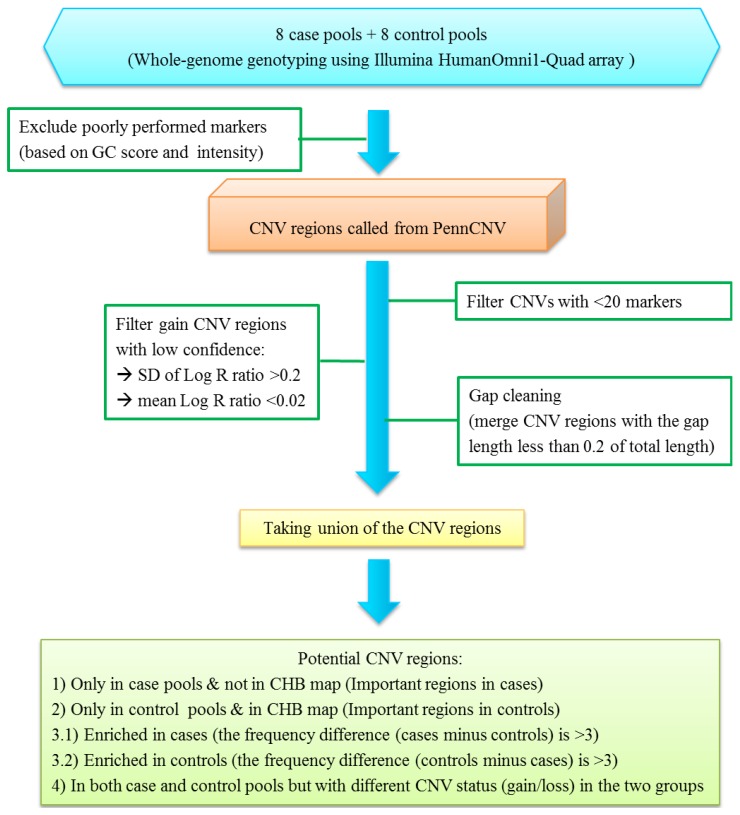
The flow chart of the criteria for copy number variation (CNV) analysis.

Figure 1 shows the flow chart of our CNV analysis. A series of quality control procedures were implemented to improve data quality before running CNV analysis. We first removed markers with missing signals in any pool or on the sex chromosomes. Markers with a genetic control (GC) score equal to zero in more than three pools were excluded. We also removed markers with a median Log R ratio >1 or <−5 across 16 pools as those outside of this threshold are likely to be false or prone to genotyping errors. The Log R ratio represents the normalized intensity of probe signals. After employing these quality control procedures, 694,475 markers were retained. We used PennCNV [9] for CNV analysis. PennCNV is a HMM-based algorithm for CNV calling, which uses normalized intensity (Log R ratio) and allele frequency data (B allele frequency) of markers to simultaneously estimate CNV region and its copy number status [9]. It could analyze array signal data from both Affymetrix and Illumina platforms. We applied this algorithm to call CNV numbers for each pool. If the copy number is equal to two, the Log R ratio is approximately zero. If the copy number is greater (a gain CNV status) or less (a loss CNV status) than two, the corresponding Log R ratio is higher or lower than zero, respectively.

The estimated CNV regions for each pool were then identified using PennCNV. We set a series of criteria to obtain informative CNV regions. First, regions with less than 20 markers were filtered out to avoid false-positive results in short regions with PennCNV analysis. A large number of gain CNV regions were predicted from 16 pools. To further reduce the likelihood of obtaining false positive findings in the gain CNV regions, we applied other criteria for the gain CNV regions by Log R ratio to increase data quality; (1) If the mean Log R ratio within the identified CNV region is less than 0.02, we filtered out this region as the intensity around zero indicating a high potential to be a normal CNV status (copy number = 2); (2) If the standard deviation of Log R ratio within the identified CNV region is greater than 0.2, we filtered out this region. The second procedure is also suggested by PennCNV for the quality control of individual samples [9]. 

Because PennCNV tends to split large CNV regions into multiple smaller regions, a merge procedure for adjacent CNV calls was applied in the next step [9]. We performed a gap cleaning procedure to merge neighboring CNVs where the ratio of the gap length and sum of the two neighboring CNV lengths is less than 0.2. At this step, we had obtained around three thousand estimated CNV regions with length ranging between 1.49 kb and 1021.08 kb in the 16 pools. To make comparisons of CNV results possible across pools, we took the union of each defined CNV region for all the pools. In total, there were 2243 unique CNV regions in case pools and 2426 unique CNV regions in control pools. In addition, different CNV calling algorithm could result in different calling results; therefore, we also used QuantiSNP [10] to analyze potential CNV regions identified by PennCNV.

For small sample size, several strategies were implemented to conduct association testing. In the present study, we first constructed the Han Chinese CNV map. We used the published CHB (Han Chinese in Beijing) CNV regions in HapMap and 2 CNV databases from Lin *et al*. [22] and Lou *et al*. [23]. We selected more informative CNVs according to the different CNV patterns between case and control pools. The informative CNVs were defined based on the following criteria.

(1) the CNV regions were only found in case pools but not in the Han Chinese CNV map; these CNVs were defined as important regions in cases;
(2) the CNV regions were only found in control pools and also reported in the Han Chinese CNV map; these CNVs were defined as important regions in controls;
(3) the CNV regions were shown in both case and control pools, but the frequency difference in the two groups is large (>3); these CNVs were defined as enriched in cases or controls;
(4) the CNV regions were found in both case and control pools, however the CNV status (gain/loss) was different in the two groups.

These selected CNV regions were potential targets for BPD and were included in the following analyses.

### 2.3. Association Testing for CNV Regions with BPD

We first conducted CNV burden analysis between case and control pools, which is employed in previous studies [24]. We conducted burden analyses stratified by CNV types (gain or loss) and size (length ≥100 kb or ≥500 kb). Secondly, using a data integration framework, we had previously built a candidate gene database for BPD, and obtained 164 prioritized susceptible loci, namely BPDgenes [25]. We mapped genes for the potential CNV regions and compared them to the BPDgenes. For the mapped genes in the CNV regions that overlapped with BPDgenes, we tested the signal differences between case and control pools. We calculated the median signal of the markers within the defined CNV regions. Both *t*-test and Wilcoxon test were used to evaluate signal associations between cases and controls. Thirdly, a functional enrichment analysis for the mapped genes in selected CNV regions was performed using WEB-based GEne SeT AnaLysis Toolkit (WebGestalt) [26]. We adopted multiple testing corrections using the Benjamini-Hochberg method for each analyzed pathway with the significance level *p* < 0.05. Finally, we searched the literature to identify previously reported CNV regions in BPD. For associated CNV regions that were found in more than one patient in previous studies, we compared them with our potential CNV regions. These results are summarized in Appendix Table A1. 

### 2.4. CNV Validation by RT-qPCR

Due to the genome-wide scale of the CNV identification, we were only able to conduct experimental validation for a few CNV regions for the proof of principle of our filtering procedures and data analysis results. Two CNV regions were selected for validation using real-time quantitative polymerase chain reaction (RT-qPCR). The first region was the 6q27 CNV (Results Section) that replicated results from two previous studies, the second was a CNV region on chromosome 3p14.2 (Results Section) showing signal differences between CNV carriers and non-carriers, including a BPD candidate gene *PTPRG*. We performed RT-qPCR using Taqman Copy number assays (Chr.3 Hs04761773_cn and Chr. 6 Hs03602538_cn) and Taqman Copy number reference assays (Applied Biosystems, Foster City, CA, USA). Individuals in the carrier case-pool and non-carrier control-pool (25 cases and 25 controls) were tested in each assay, and the RT-qPCR was carried out in triplicate. Sequence Detection Software (SDS) was used for exporting the threshold cycle (Ct) data and further analyzing differences in Ct values (ΔCt) between the test locus and the control locus. Copy number variation was analyzed with the CopyCaller software [27]. We used Student’s *t*-test to compare raw copy number signals calculated from ∆Ct values to determine the statistical significance of predicted copy-number differences in cases and controls. The significant threshold was defined by *p* < 0.05. 

## 3. Results

Results of the CNV burden analysis for the potential CNV regions are displayed in Table 1. There was a relatively higher proportion of loss CNV regions (≥100 kb) in BPD patients than in controls, though the difference did not reach statistical significance (Wilcoxon *p*-value = 0.105). There was no significant difference in other types of the CNVs between BPD cases and controls.

Table 2 shows the results of gene mapping in the selected CNV regions. There were 882 CNV regions that were only found in case pools and not mapped to the Han Chinese CNV map (Important regions in cases). In contrast, 94 CNV regions were only found in control pools and were mapped to the Han Chinese CNV map (Important regions in controls). In addition, two CNV regions were enriched in cases for which the frequency of case pools having this CNV is three more than that in control pools, and 26 CNV regions were enriched in controls. Only one CNV region had a different CNV status, with loss status in cases and gain status in controls. In total, 1247 genes were mapped to these selected CNV regions, and 30 of them were overlapped with the prioritized genes in the BPDgenes [25]. We compared the CNV signal differences between cases and controls and focus on the regions that had affected genes mapped to the BPDgenes (*i.e.*, 30 CNV regions). We presented the CNV regions that exhibited signal differences between cases and controls with *p*-value <0.2 using *t*-test or Wilcoxon test, with these CNVs regarded as the top priority regions for BPD in our samples (see Table 3). Two of the CNV regions showed a significant difference (at *p* < 0.05) using either a *t*-test or Wilcoxon test. This included CNV regions on chromosomes 11 and 22.

Functional enrichment analysis was performed for the 1247 CNV genes to explore their biological information. These mapped genes were significantly enriched (adjusted *p*-value less than 0.05) in 21 GO terms (see Table 4). In addition, the enrichment analysis was conducted using the database of disease associated genes in WebGestalt. Two disease categories (bipolar disorder and mood disorder) reached statistical significance (*p* < 0.05) (Appendix Table A2).

**Table 1:** CNV burden analysis in BPD case pools and control pools.

CNV size	CNV	Sample	No. of	Mean CNVs	Wilcoxon
	type	Group	unique CNVs	per pool	*p*-value
≥100 kb	Both	Controls	1441	346.25	0.645
		Patients	1446	307.25	
≥100 kb	Gain	Controls	1438	345.5	0.645
		Patients	1434	304.375	
≥100 kb	Loss	Controls	3	0.75	0.105
		Patients	12	2.875	
≥500 kb	Both	Controls	43	16	0.798
		Patients	44	15.5	
≥500 kb	Gain	Controls	43	16	0.798
		Patients	44	15.5	
≥500 kb	Loss	Controls	0	0	NA
		Patients	0	0	

Abbreviation: CNV, copy number variation; BPD, Bipolar Disorder.

Lastly, we compared our CNV results to findings in the previous studies of BPD [7,24,28,29,30,31,32,33]. We found that five of the 1005 selected CNV regions (6q16.3, 6q27, 9q34.3, and two regions on 19p12) were also identified in previous studies (see Table 5). All of the overlapped regions were only found in cases. Four were gain CNV regions, and one region on 19p12 was loss CNV status. Priebe *et al*. found a CNV region at 6q27 that was overrepresented in bipolar patients with age at onset ≤21 [28]. This region was also reported to be enriched in affected members of a three-generation Amish pedigree of European descent [29]. Two CNV regions on chromosome 19 were both located at 19p12. Grozeva *et al*. found that these CNV regions were associated with BPD in Wellcome Trust Case Control Consortium samples [30]. In addition, Bergen *et al*. reported a duplication CNV region (9q34.3) in 14 patients with BPD, 17 patients with schizophrenia, and 11 normal controls [31]. Association testing of this region was significant when combining the two patient groups. Moreover, McQuillin *et al*. reported CNV regions at 6q16.3 in 2 BPD cases in British samples [32]. The rest of the 1000 CNV regions were not reported in more than one individual in previous studies.

**Table 2:** Potential CNV regions related to bipolar disorder and the information of mapping genes.

Potential CNV regions	No. of CNV (Gain/Loss)	Mean CNV length (kb)	No. of mapped Genes in CNV regions ^a^	Genes overlapped with the list in BPDgenes ^b^
Important regions in cases	882 (859/23)	120.52	982	*ANK3*, *ARNTL*, *ASTN2*, *CHST11*, *CSMD2*, *DACH*, *DLG2*, *DPP10*, *DSCAM*, *GRIK1*, *HTR6*, *KALRN*, *MCTP1*, *MYO3B*, *NALCN*, *NOS1*, *OPCML*, *OR6S1*, *PARK2*, *PDLIM5*, *PLCB1*, *PTPRG*, *SLC39A3*, *SYN3*, *TGFB2*, *UGT1A10*, *VAV3*
Important regions in controls	94 (94/0)	91.36	164	*DMGDH*
Regions enriched in cases	2 (2/0)	447.74	0	None
Regions enriched in controls	26 (25/1)	244.49	86	*CSMD2*, *OPCML*
Different CNV status in cases and controls	(1 Gain CNV in 1 control /1 Loss CNV in 1 case)	253.96	15	None

^a^ Genes that are partial or fully included in the CNV regions; ^b^ 164 prioritized loci for BPD in the BPDgenes [25].

**Table 3:** Signal differences between case and control pools for identified CNV regions.

Chr	Position ^a^	CNV type ^b^	Length (kb)	Affected Genes ^c^	*p*-value (*t* test)	*p*-value (Wilcoxon test)
1	34,268,681–34,936,979	Gain in 6 controls and 2 cases	668.30	***CSMD2***, *C1orf94*	0.192	0.169
3	61,681,785–61,928,141	Gain in 1 case	246.36	***PTPRG***	0.171	0.234
4	95,487,295–95,868,284	Gain in 3 case	380.99	***PDLIM5***, *ENH*, *ENH1*, *LIM*	0.646	0.161
9	118,292,450–118,450,577	Gain in 1 case	158.13	***ASTN2***, *KIAA0634*, *bA67K19.1*	0.157	0.169
11	13,224,130–13,256,233	Gain in 1 case	32.10	***ARNTL***, *BMAL1*, *BMAL1c*, *JAP3*, *MGC47515*, *MOP3*, *PASD3*, *TIC*, *bHLHe5*	0.007 *	0.010 *
12	103,611,282–103,669,104	Gain in 1 case	57.82	***CHST11***, *C4ST*, *C4ST-1*, *C4ST1*, *DKFZp667A035*, *FLJ41682*, *HSA269537*	0.113	0.065
22	31,480,536–31,564,931	Gain in 1 case	84.40	***SYN3***, *TIMP3*	0.029 *	0.021 *

Abbreviation: Chr, Chromosome; ^a^ Position were assembled by NCBI build 36 (UCSC hg 18); ^b^ Gain or loss CNV type in case or control pools; ^c^ The bold labels the affected Genes in the CNV regions that are mapped to BPDgenes [25]; * *p*-value is smaller than 0.05.

In experimental validation of CNV at chromosome 3p14.2, two BPD individuals and one control subject out of 25 cases and 25 controls were found to have gain CNV at PRPRG region. The signal intensity of copy number value in the gain CNV that compares with a CNV status of 2 was 2.52 ± 0.002 (mean ± SD) versus 1.98 ± 0.033 (mean ± SD). The difference reached statistical significance (*p* < 0.05) using *t* test. Thus, confirmatory RT-qPCR experiments lent further support for this CNV validation. For the chromosome 6q27 region, this region was overlapped with CNV findings reported in previous BPD studies but did not show signal difference between our cases and controls. Experimental results showed that no individual (out of 25 cases and 25 controls) was validated to have gain CNV. The signal intensity of copy number value in cases and controls was 1.99 ± 0.03 and 1.92 ± 0.05, respectively.

**Table 4:** Gene Set Enrichment Analysis of genes mapped to potential CNV regions.

Enriched GO category	Database ID	*p*-value ^a^	Adjusted *p*-value ^b^	O ^c^	N ^d^
**Biological process**					
biological adhesion	GO:0022610	1.16 × 10^−8^	1.5 × 10^−5^	93	905
cell adhesion	GO:0007155	2.13 × 10^−8^	1.5 × 10^−5^	92	903
cell-cell adhesion	GO:0016337	4.03 × 10^−5^	0.0190	41	374
**Cellular component**					
neuron projection	GO:0043005	1.03 × 10^−7^	2.2 × 10^−5^	69	628
synapse	GO:0045202	1.13 × 10^−5^	0.0008	51	478
cell projection	GO:0042995	9.19 × 10^−6^	0.0008	102	1173
axon	GO:0030424	1.85 × 10^−5^	0.0010	34	276
dendrite	GO:0030425	2.61 × 10^−5^	0.0011	39	341
cell projection part	GO:0044463	4.52 × 10^−5^	0.0016	59	610
synaptic membrane	GO:0097060	0.0001	0.0031	26	208
synapse part	GO:0044456	0.0002	0.0053	38	361
neuron spine	GO:0044309	0.0004	0.0086	20	153
dendritic spine	GO:0043197	0.0004	0.0086	20	153
cell periphery	GO:0071944	0.0010	0.0195	267	3989
keratin filament	GO:0045095	0.0013	0.0227	10	57
plasma membrane	GO:0005886	0.0017	0.0227	260	3905
postsynaptic density	GO:0014069	0.0016	0.0227	15	111
cytoskeleton	GO:0005856	0.0017	0.0227	119	1613
dendritic spine head	GO:0044327	0.0016	0.0227	15	111
postsynaptic membrane	GO:0045211	0.0028	0.0333	20	178
presynaptic membrane	GO:0042734	0.0028	0.0333	9	53

^a^ *p*-value was derived from Fisher’s exact test; ^b^ *p*-value was adjusted by Benjamini-Hochberg method; ^c^ O: number of genes in the gene set and also in the GO category; ^d^ N: number of reference genes in the pathway category.

**Table 5:** Comparison of our CNV results of bipolar disorder with findings in previous studies.

Present Study						Previous Studies			
Location	Position ^a^	CNV type	Length (kb)	Affected Genes	No. of cases/controls	Position ^a^	CNV type	Length (kb)	References
*6q16.3*	101966969:102040222	Gain	73.250	GRIK2	1/0	101953625:102624651	Unknown	671.027	[32]
*6q27*	168320777:168376820	Gain	56.044	KIF25, FERM,	1/0	168090000:168330000	Gain	240.000	[28,29]
				MILT4, DACT2					
*9q34.3*	138149942:138217164	Gain	67.233	None	2/0	136600001:140273252	Gain	3673.252	[31]
*19p12*	20091264:2029165	Gain	200.402	ZNF682, ZNF90,	1/0	20001614:20177979	Gain/Loss	176.365	[30]
				ZNF486					
*19p12*	24193894:24282139	Loss	88.246	ZNF254	2/0	24013968-24295825	Gain	281.857	[30]

^a^ Position were assembled by NCBI build 36 (UCSC hg 18).

## 4. Discussion

The current study applied a series of filtering and data analysis procedures to identify CNV regions that are related to bipolar disorder using a DNA pooling strategy. Several filtering methods have previously been developed for array data to increase detection power, in particular for microarray gene expression data. A screening threshold is usually set based on the variance of expression signals, where probes with low variance are excluded as non-informative markers [34,35]. The filtering procedures become even more important while adopting a DNA pooling strategy. In a genome-wide association study using pooled DNA, SNP quality control filters are set based on the indicators calculated from pooled intensity [36]. Similar concepts are adopted in our filtering scheme for pooling CNV data. The criteria we set for a CNV analysis with small sample size could assist for CNV identification by reducing the potential impact of experimental noise to explore the relationships between CNVs and the trait of interest. A number of potential CNV regions are reported for BPD in our Han Chinese samples that may warrant further investigation. 

Higher CNV burden is often observed in patients with psychiatric disorders when compared with healthy controls [37]. However, the reported specific CNV regions, even in large-scale Caucasian samples, are rarely replicated [24,28,30,32]. In the present study, we found that the burden of loss status CNVs in BPD cases is higher than in controls, though the comparisons did not reach statistical significance. Similar findings are reported in Zhang *et al*., which conducted a genome-wide CNV study of BPD in European Americans. They found that the number of singleton deletion CNVs in BPD cases is significantly higher than those in controls (*p* = 0.007) [38]. Other studies reported fewer CNVs with loss status in BPD cases than controls. For instance, in a young adult British sample, McQuillin *et al*. found that BPD subjects have significantly fewer deletion CNVs, with the size ranging from 200–500 kb compared to controls (*p* = 0.039), while fewer singleton duplication CNVs with the size over 100 kb are also found in BPD cases (*p* = 0.03) [32]. In addition, large (≥500 kb) inherited duplication CNVs are also found to be enriched in familial BPD cases (*p* = 0.03) [24]. The distinct findings of excessive deletion or duplication CNVs among BPD patients in the previous studies may result from the differences in sample populations, clinical characteristics of BPD cases, the CNV detection platforms, and CNV analysis criteria. Some studies advocate to subgroup BPD patients to obtain genetically more homogeneous groups. Age at onset is an often considered feature. Two CNV studies divided BPD cases into early or late onset subgroups by the age of onset (AO) of BPD diagnosis [24,28]. Comparing with healthy controls, one study reported that the rate of *de novo* CNVs is significantly higher in the patients group with AO ≤ 18 [24], whilst the other reported a higher frequency of microduplication CNVs in patients with AO ≤ 21 [28]. Two regions with duplication CNVs—the 6q27 and 10q11 CNV regions—are especially noted for early onset BPD [28]. To stratify BPD patients into subgroups based on relevant clinical characteristics could be considered in future CNV studies to reduce heterogeneity among patients.

Through a series of data analysis procedures and a comprehensive literature search, we identified several CNV regions in relation to BPD. Some of the regions are reported in previous CNV studies, and some are novel regions. Novel CNV regions may be ethnic group specific and provide additional clues for exploring the pathogenesis of BPD in Han Chinese population. At the first stage of data screening, we found approximately 1000 novel CNV regions. Using a gene prioritization framework, we had previously built a gene database for BPD. The top list in the BPDgenes has a higher combined score, and thus higher confidence to be associated with bipolar illness. There are 30 genes in our identified CNV regions that are mapped to the BPDgenes (see Table 2), and the CNV regions that encompass these genes are considered high priority for further association testing. We reported signal differences between cases and controls for CNV regions on several chromosomes (see Table 3). These CNV regions have a higher potential to be related with BPD, and genes mapped to these regions are candidate genes for BPD. For instance, *ARNTL* is a circadian gene and has been found to be associated with BPD in Caucasian samples [39,40]. Gene *PTPRG* has previously been found to be associated with schizoaffective disorder [41]. We conducted RT-qPCR experimental validation for the *PTPRG* gene region, and the gain CNV status was validated, for which the signal intensity was higher in BPD cases than in controls. Research on the functional properties of these affected genes in potential CNV regions and how they link to the etiology of BPD may help point to a direction for the development of a new drug target.

In addition to novel regions, there are five CNV regions (located at 6q16.3, 6q27, 9q34.3, 19p12) overlapped with the results from previous studies (see Table 5). One CNV region, 6q16.3, is reported to be associated with BPD in both ours and the study conducted by McQuillin *et al*. [32]. Gene *GRIK2* is mapped to this CNV region. This gene is essential for brain development [42]. Previously, polymorphisms in *GRIK2* gene have been reported to exhibit associations with obsessive-compulsive disorder [43] and autism spectrum disorders [44]. A loss CNV was found in our cases in 19p12 while a gain CNV in the same region was reported in Grozeva *et al*. [30]. Olsen *et al*. conducted a meta-analysis for three CNV regions—6q27 and 19p12 (two CNVs)—that are overrepresented in patients with affective disorder in three case-control studies [45]. However, the association testing is not significant for the three CNVs. As very few individuals possess either of these CNVs, a reliable test is not easy to perform for testing CNV associations with disease outcomes. Further replicated studies with larger sample size are needed to verify the relationship between candidate CNVs and BPD. 

The heterogeneity of genetic architecture across populations often leads to diverse genetic findings on the phenotypic outcomes of interest [46,47]. The diversity of CNVs in different ethnic groups has also been noted previously [48,49]. Among CNV studies in BPD, to our best knowledge, we are the first to conduct a genome-wide level of CNV analysis in an Asian population. For identified potential CNV regions for BPD, we also compared our results with findings from previous studies in different samples. The CNV regions that reported consistently in ours and previous studies may represent common risk regions across populations for BPD. If validated by experiments, novel CNV regions that were only reported in our study may indicate population specific genetic components for BPD, such as the CNV region on chromosome 3p14.2.

By conducting functional analysis using GO terms for our mapped CNV genes, we found that the top three enriched pathways were involved in biological adhesion, cell adhesion, and neuron projection (Table 4). Several other genetic association studies, but not CNV studies, have also performed pathway analysis for BPD related candidate genes. The top significant GO pathways reported in Chang *et al*., are amine binding, synapse transmission, and transmission of nerve impulse [50]. Another study applied pathway analysis while incorporating information of allele-specific gene methylation [51]. They reported enriched pathways for extracellular matrix in brain, gated ion channel, and neurotransmitter receptor related pathways. Their findings support the involvement of biological functions of cell adhesion and neuronal transmission underlying bipolar illness. Further studies to investigate the interaction and networks among identified molecules for BPD could be conducted to understand the pathophysiology of BPD. 

There are several limitations in the present study. First, DNA pooling strategy is restricted to the original study design (*i.e.*, for our study, bipolar disorder *vs*. control) and not flexible for conducting secondary data analysis. If there is a belief of true genetic heterogeneity in disease subtypes or the genetic factors are influenced by other covariates, it is not possible to adjust results for these concerns. Second, employing a series of data filtering steps may cause false-negative findings for certain CNV regions. In addition, because there is no consensus of a standard method for CNV calling, we used a second calling algorithm for our identified CNV regions. In the 12 reported CNV regions (listed Table 3 and Table 5), only the loss CNV region at chr19: 24193894:24282139 were consistently called by both calling algorithms. It is consistent with prior study showing that both algorithms have high reproducibility rates in loss CNV regions, but low rates in gain CNV regions [52]. In future study, applying multiple CNV calling algorithms and conducting experimental validation are desired. Third, due to having a small sample size we could only identify relatively common CNVs. Very rare or *de novo* CNVs are likely to be ignored. Nevertheless, we conducted power calculation [53] using median Log R ratio within a CNV region. We took one CNV at chr11: 13,224,130–13,256,233 as an example (see Table 3). The power for detection of signal difference between cases and controls can reach 0.87 in the current study. Follow-up individual studies with larger sample sizes should be designed to validate and test associations for identified CNV regions. Fourth, CNV regions that do not have any mapped genes (*i.e.*, in gene desert regions) are not reported as the potential roles of these CNVs are not clear. Lastly, the experimental quality to detect CNV signals is a concern for pooling based design as there are no easy indicators to estimate the accuracy of CNV signal intensities. Other than two selected CNVs for experimental validation, we do not conduct a genome-wide level of validation and independent replication studies for our identified CNV regions. Further large-scale individual and replication studies are needed to investigate the roles of these CNVs and eventually provide clues for the underlying mechanisms of bipolar illness.

## 5. Conclusions

There are many difficulties faced in performing CNV studies as most of the disease associated CNVs are complex, rare and usually with marginal effect size. The heterogeneity of bipolar disorder brings another challenge in explaining the CNV results. Proper data filtering and analysis strategies are recommended in exploring the relationships between CNVs and the trait of interest. We conducted the first pilot study of CNV association with BPD in Han Chinese population and identified several potential CNV regions for BPD. It is important to design further validation experiments and perform basic research for these CNVs to reveal their biological roles and explain their involvement in bipolar illness.

## Appendices

**Appendix Table A1:** Reported associated CNV regions in previous BPD studies.

No.	Chromosome	Start Position	End Position	CNV type	Length (kb)	Gene	References
1 *	6q27	168,090,000	168,330,000	duplication	240	*KIF25*, *FERM*, *MILT4*, *DACT2*	[28,29]
2 *	6q16.3	101,953,625	102,624,651	unknown	671.027	*GRIK2*	[32]
3 *	9q34.3	136,600,001	14,0273,252	duplication	3,673.252	None	[31]
4 *	19p12	20,001,614	20,177,979	both	176.366	*ZNF682, ZNF90, ZNF486*	[30]
5 *	19p12	24,013,968	24,295,825	duplication	281.858	*ZNF254*	[30]
6	1	28,399,376	28,842,172	unknown	442.797	*DNAJC8*, *ATPIF1*, *SESN2*, *MED18*, *SNHG3-RCC1*, *RCC1*, *TRSPAP1*, *RAB42*, *TAF12PHACTR4*	[32]
7	1	47,415,160	47,600,013	duplication	184.854	*PDZK1IP1*; *TAL1*; *STIL*; *CMPK1*	[24]
8	1	144,439,082	144,791,590	unknown	352.509	*PDZK1*, *GPR89A*, *GPR89C*, *NBPF11*, *LOC728912*, *FAM108A3*	[32]
9	1q21.1	142,400,001	148,000,000	both	5,600	None	[31]
10	1q25.1	173,769,777	173,978,862	duplication	209.086	*TNR*	[30]
11	1	232,723,219	232,828,069	unknown	104.851	*IRF2BP2*	[31]
12	2	196,772,221	197,165,580	unknown	393.36	*HECW2*	[32]
13	3	8,896,559	8,980,146	unknown	83.588	*RAD18*	[32]
14	3p14	65,649,762	65,848,146	deletion	198.385	*MAGI1*	[33]
15	3p26	2,124,587	2,955,648	duplication	831.062	*CNTN4*	[24]
16	3q	120,920,000	121,100,000	deletion	180.001	*GSK3beta*	[7]
17	4q34.3	180,892,619	180,921,485	unknown	28.867	None	[31]
18	5	180,098,728	180,099,664	unknown	0.937	*OR2Y1*	[32]
19	6	56,430,743	56,816,422	unknown	385.68	*DST*	[32]
20	6	57,290,380	57,621,335	unknown	330.956	*PRIM2*	[32]
21	6	157,140,777	157,572,094	unknown	431.318	*ARID1B*	[32]
22	7	34,935,017	35,044,178	unknown	109.162	*DPY19L1*	[32]
23	7	75,975,221	76,052,734	unknown	77.514	*UPK3B*	[32]
24	7	88,226,688	89,777,622	unknown	1,550.94	*ZNF804B*, *MGC26647*, *STEAP1*, *STEAP2*, *FLJ21062*	[32]
25	7	132,588,362	133,401,053	unknown	812.692	*EXOC4*	[32]
26	8	13,236,908	13,304,907	unknown	68	*DLC1*	[31]
27	9	111,037	169,075	unknown	58.039	*CBWD1*	[32]
28	9	71,289,871	71,308,782	duplication	18.912	None	[29]
29	9	134,871,014	134,890,520	unknown	19.507	*GTF3C5*, *GFI1B*	[31]
30	9q31.1	104,826,097	104,885,068	both	58.972	None	[30]
31	10	8,108,359	8,192,845	duplication	84.487	*GATA3*	[24]
32	10q11	47,010,000	47,170,000	duplication	160	*ANTXRL*	[28]
33	10	50,334,496	50,490,772	unknown	156.277	*ERCC6*, *PGBD3*, *CHAT*, *SLC18A3*	[32]
34	10	51,497,689	52,053,743	unknown	556.055	*FAM21A*, *FAM21B*, *ASAH2*, *SGMS1*	[32]
35	12	7,884,583	8,017,012	duplication	132.43	*SCL2A3M*, *SLC2A14*	[29]
36	12p11.21	31,202,250	31,301,551	duplication	99.302	*OVOS2*	[30]
37	12	107,243,140	107,266,950	unknown	23.811	*CMKLR1*	[31]
38	13	49,932,650	49,982,221	deletion	49.572	*AJ412031*; *AJ412041*	[24]
39	13	90,848,887	92,317,488	unknown	1,468.60	*GPC5*	[32]
40	14	24,044,551	24,047,311	unknown	2.761	*CMA1*	[32]
41	15q.2	21,905,523	22,023,095	deletion	117.573	None	[29]
42	15q13.2	28,000,001	29,000,000	both	1,000	*CHRFAM7A*, *MRMR15*	[31]
43	16p13.11	14,700,001	16,700,000	duplication	2,000	None	[31]
44	16	15,435,825	15,889,948	unknown	454.124	*C16orf45*, *KIAA0430*, *NDE1*, *MYH11*, *C16orf63*	[32]
45	16	15,950,934	16,296,168	unknown	345.235	*ABCC1*, *ABCC6*, *NOMO3,*	[32]
46	16	16,333,234	16351940	unknown	18.707	*LOC339047*	[32]
47	16	68,705,029	69,071,678	unknown	366.65	*PDPR*, *MGC34761*, *EXOSC6*, *AARS*, *DDX19B*, *DDX19A*, *ST3GAL2*, *FUK*	[32]
48	17	36,465,156	36,477,177	deletion	12.022	*KRTAP2-4*; *KRTAP2-4*	[24]
49	17q25.1	68,400,001	72,200,000	duplication	3800	None	[31]
50	18p11.21-11.1	14,694,694	15,092,421	duplication	397.728	*ANKRD30B*	[30]
51	18	27,210,737	27,312,663	unknown	101.927	*DSG4*, *DSG3*	[32]
52	19	49,581,647	49,644,505	unknown	62.859	*ZNF285A*, *ZNF229*	[32]
53	19	58,644,961	58,689,358	unknown	44.398	*ZNF761*, *ZNF813*, *ZNF765*, *ZNF331*	[32]
54	21q11.2	13,200,001	15,300,000	both	2,100	*ANKRD21*, *LOC441956*, *LIPI*, *RBM11*	[31]
55	21	36,429,132	36,440,730	unknown	11.599	*CBR3*	[32]

***** No. 1–5 CNV regions were overlapped with our findings.

**Appendix Table A2:** Enriched gene set of the mapped genes in the potential CNV regions.

Functional Category	Genes on CNV	*p*-value ^c^	Adjusted *p*-value ^d^	O ^a^	N ^b^
**Bipolar Disorder** **(DB_ID:PA447199)**	*PCDH17*, *ANK3*, *GABRR3*, *GABRG2*, *NOS1*, *CNTNAP2*, *ADCYAP1*, *PTPRG*, *NRXN1*, *PCLO*, *TACR1*, *TCF4*, *JMJD8*, *ADCY3*, *CSMD1*, *DPP10*, *CNTN5*, *RELN*, *NALCN*, *HTR5A*, *AGAP1*, *DFNB31*, *HTR4*, *GPC6*, *ATP8A2*, *GABRA1*, *CNTN6*, *ASTN2*, *FAT1*, *ADCY8*, *ARNTL*, *RPL14*, *PPP3CC*, *NRG1*, *MAGI1*, *PDLIM5*, *MMP16*, *HTR2A*, *CHRM2*	2.40 × 10 ^−5^	0.0252	39	286
**Mood Disorders** **(DB_ID:PA447209)**	*ANK3*, *GABRR3*, *GABRG2*, *NOS1*, *SST*, *CNTNAP2*, *ADCYAP1*, *PTPRG*, *PCLO*, *GRM5*, *TACR1*, *TCF4*, *HTR6*, *DPP10*, *CNR2*, *CNTN5*, *GPM6A*, *NALCN*, *RELN*, *HTR5A*, *GRIK1*, *DFNB31*, *AGAP1*, *CNTN6*, *GABRA1*, *ASTN2*, *NXPH1*, *FAT1*, *ARNTL*, *NRG1*, *PDLIM5*, *HTR2A*, *CHRM2*	5.53 × 10 ^−5^	0.0290	33	235

^a^ O: number of genes in the gene set and also in the category; ^b^ N: number of reference genes in the category; ^c^ *p*-value was derived from Fisher’s exact test; ^d^ adjusted-*p*-value was corrected by Benjamini-Hochberg correction.
